# Model Uracil-Rich RNAs and Membrane Protein mRNAs Interact Specifically with Cold Shock Proteins in *Escherichia coli*

**DOI:** 10.1371/journal.pone.0134413

**Published:** 2015-07-30

**Authors:** Daniel Benhalevy, Elena S. Bochkareva, Ido Biran, Eitan Bibi

**Affiliations:** Department of Biological Chemistry, Weizmann Institute of Science, Rehovot 76100, Israel; Univ. of Edinburgh, UNITED KINGDOM

## Abstract

Are integral membrane protein-encoding mRNAs (MPRs) different from other mRNAs such as those encoding cytosolic mRNAs (CPRs)? This is implied from the emerging concept that MPRs are specifically recognized and delivered to membrane-bound ribosomes in a translation-independent manner. MPRs might be recognized through uracil-rich segments that encode hydrophobic transmembrane helices. To investigate this hypothesis, we designed DNA sequences encoding model untranslatable transcripts that mimic MPRs or CPRs. By utilizing *in vitro*-synthesized biotinylated RNAs mixed with *Escherichia coli* extracts, we identified a highly specific interaction that takes place between transcripts that mimic MPRs and the cold shock proteins CspE and CspC, which are normally expressed under physiological conditions. Co-purification studies with *E*. *coli* expressing 6His-tagged CspE or CspC confirmed that the specific interaction occurs *in vivo* not only with the model uracil-rich untranslatable transcripts but also with endogenous MPRs. Our results suggest that the evolutionarily conserved cold shock proteins may have a role, possibly as promiscuous chaperons, in the biogenesis of MPRs.

## Introduction

In addition to encoding proteins, mRNAs harbor information required for controlling post-transcriptional regulatory pathways, such as processing, translation, degradation, and cellular localization [1, 2]. For selective cellular localization, mRNAs utilize various protein-interaction determinants (structural, sequence-specific, or nonspecific) [3], mostly in 3’ untranslated regions, although unique exceptions have also been described [e.g. [4]]. In this regard, *E*. *coli* represents an interesting case because unlike in eukaryotes [5] the prokaryotic mRNAs usually do not contain 3’ regulatory untranslated sequences (Daniel Dar and Rotem Sorek, personal communcation), suggesting that they might be recognized through their relatively short 5’ untranslated regions or through their open reading frames. mRNAs that encode integral membrane proteins (IMPs) across evolution are usually translated at distinct membrane locations, and our studies in *E*. *coli* have suggested a step through which IMP-encoding mRNAs (MPRs) are selectively targeted to membrane-bound ribosomes [6–8]. This proposal of translation-independent mRNA targeting to membranes has become an emerging concept in cell biology, based on a growing body of direct experimental evidences in eukaryotes and prokaryotes [9–13]. However, the question of how such a large and diverse group of transcripts can be specifically recognized has not yet been resolved. This question is even more challenging if the recognition determinants reside within open reading frames.

The question whether MPRs are specifically recognized in the cell is generally important and challenging, regardless of the targeting concept described above. In the past, we utilized bioinformatics and revealed an exceptionally strong uracil (U) bias in the coding sequences of MPRs [14]. We observed that these transcripts are characterized by ~60 nucleotide-long U-rich stretches that encode ~20 amino acid-long transmembrane helices.

In the present work, as a first step toward examining the hypothesis that U-richness is responsible for recognizing MPRs, we searched for proteins that bind such RNAs specifically in *E*. *coli*. To this end, we designed and used synthetic untranslatable U-rich transcripts that mimic MPRs in order to search for proteins that interact specifically with these RNAs. Our results show that U-rich RNAs, including endogenous MPRs, interact specifically with a group of cold shock proteins, which are normally expressed in *E*. *coli* at a physiological temperature.

## Results and Discussion

### Construction and characterization of model untranslatable transcripts

**Fig 1:**
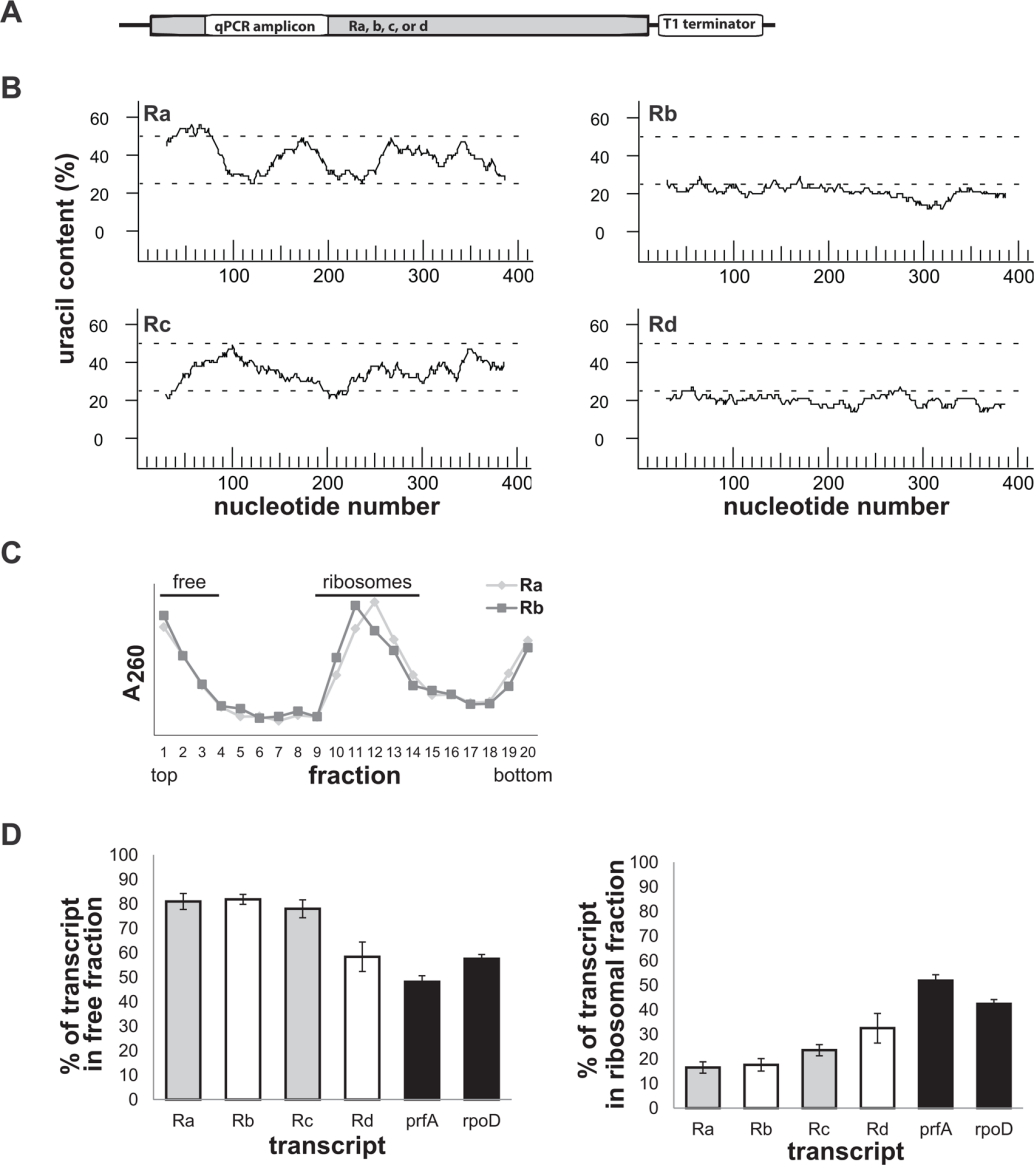
Characterization of 4 model untranslatable RNAs. **(A)** Schematic representation of the Ra-Rd encoding genes [see text, S1 Fig]. **(B)** Uracil content of the model transcripts, utilizing a sliding window of 55 nucleotides as calculated by the software DNA Strider. **(C)** Wild type *E*. *coli* expressing Ra or Rb were disrupted by sonication and cell extracts were fractionated by sucrose density gradient (a representative gradient is shown). The gradient fractions were analyzed for RNA content (A_260_). The indicated free and ribosomal fractions were pooled. **(D)** The contents of the indicated R transcripts and endogenous, translatable transcripts encoding PrfA and RpoD as controls, were measured by qPCR in the pooled free and ribosomal fractions. Error bars indicate SEM (n = 3).

In order to determine whether U-rich mRNAs bind specific factors in a translation-independent manner, we constructed 4 plasmids, each harboring a different 414-bp-long DNA fragment that encodes a model untranslatable RNA (Ra-Rd, Fig 1A and 1B; S1 Fig). Ra mimics part of the IMP *lacY* open reading frame, encoding its N-terminal 138 amino acids, which includes 4 transmembrane helices (TMs). Rb mimics part of the cytoplasmic protein *lacZ* ORF, which includes amino acids 336–473. Similarly, Rc and Rd are derived from genes encoding part of the IMP MelB and the cytoplasmic protein MelA, respectively. All the 4 transcripts are devoid of genuine ribosome binding sites and in addition, they are decorated with stop codons and contain no start codons (S1 Fig). As such, these transcripts are considered untranslatable and allow investigation of their translation-independent interactions (see later).

Next, to examine our design and determine whether these RNAs engage ribosomes *in vivo*, we expressed the R transcripts in wild-type *E*. *coli* and investigated their distribution by sucrose gradient fractionation (Fig 1C), RNA extraction, and qPCR analysis (Fig 1D). As a control, we examined the distribution of endogenous mRNAs encoding PrfA and RpoD. The results indicate that regardless of their U-richness, all the R transcripts were overrepresented in the ribosome-free fraction, in contrast to the endogenous mRNA, which were less overrepresented in this fraction.

### *In vitro* synthesized U-rich RNAs interact specifically with cold shock proteins (CSPs)

**Fig 2:**
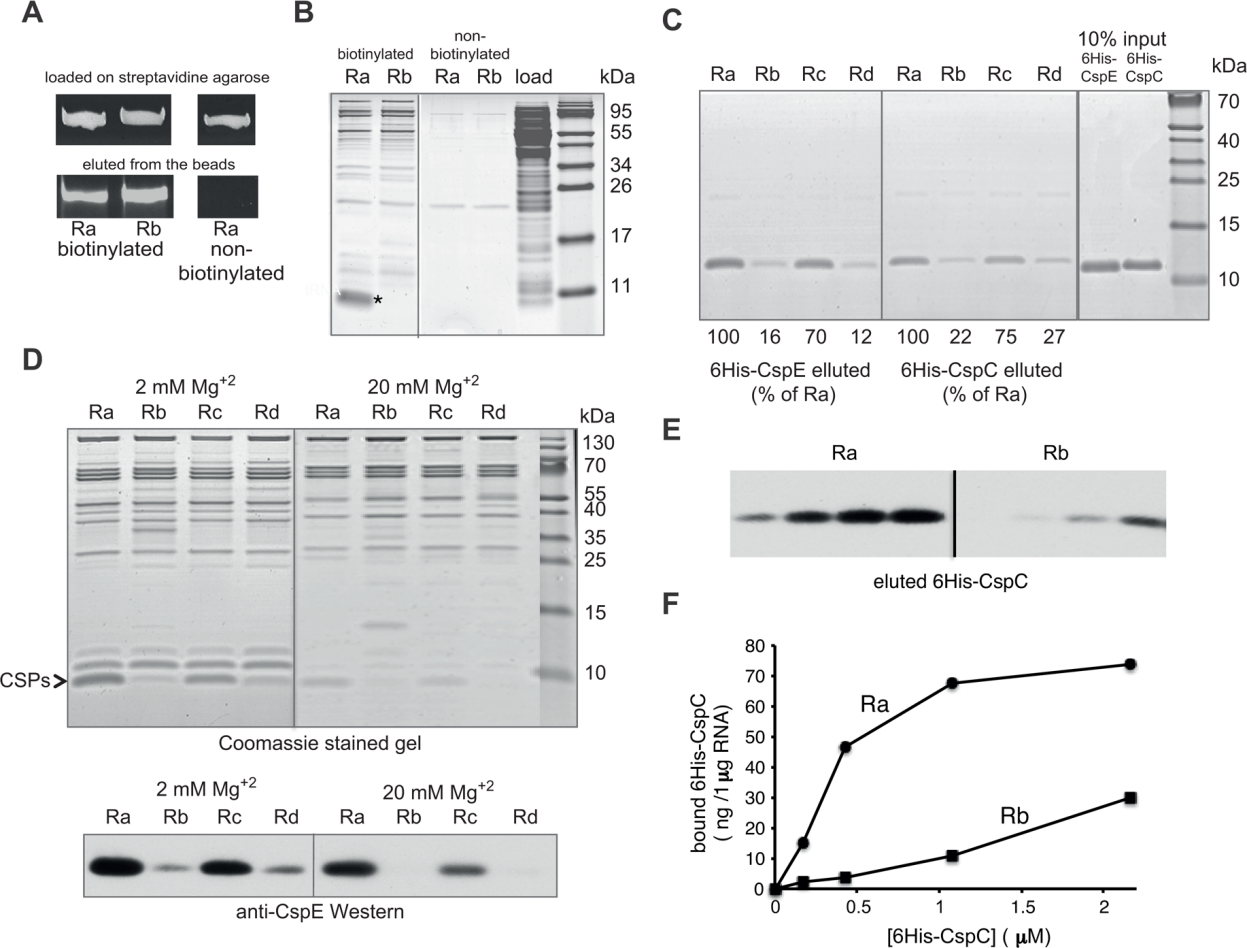
Identification of protein-U-rich RNA interactions. Ra and Rb were synthesized *in vitro* with or without biotinylated CTP. The RNAs were incubated with *E*. *coli* extract (S300) in the presence of 15 mM MgCl_2_ and then trapped by streptavidin beads. After extensive wash, the bound material was released in urea and SDS-containing buffer, and analyzed by **(A)** RNA-PAGE, stained with ethidium bromide and **(B)** protein Tris-tricine SDS-PAGE, stained with Instant blue. (*) Indicates specific U-rich RNA-binding proteins. These proteins were identified by mass spectrometry as CspE and CspC. **(C)** Biotinylated Ra-Rd were incubated with purified 6His-CspE or 6His-CspC in the presence of 15 mM Mg^2+^. RNA-protein complexes were trapped by streptavidin beads, eluted with SDS, separated by tris-tricine SDS-PAGE, and the gels were stained with Instant blue. Quantitation was performed by densitometry and expressed as percentage of the proteins that were eluted with Ra. **(D)** *In vitro* synthesized biotinylated Ra-Rd were incubated with an *E*. *coli* S300 fraction in the presence of 2 or 20 mM Mg^+2^. Uper panel, Eluted material was separated by tris-tricine SDS-PAGE and stained with instant blue. Lower panel, Western blot analysis of the eluted material with anti-CspE antibodies. **(E)** Biotinylated Ra and Rb (0.17 μM) were incubated with increasing concentrations of purified 6His-CspC (0.17–2.2 μM). RNA-protein complexes were trapped by streptavidin beads, and after wash treated with RNaseA and eluted with SDS buffer (see methods). Protein samples containing the same amount of RNA were separated by tris-tricine SDS-PAGE, and analyzed by Western blotting with anti CspE antibodies. **(F)** Quantitation of protein bands shown in **E**.

To address the question whether and how U-rich RNAs are specifically recognized in the cell by additional factors in a translation-independent manner, we searched for cytosolic proteins that might interact specifically with Ra but not with Rb. To this end, we synthesized Ra and Rb *in vitro* in the presence of a biotinylated cytosine tri-phosphate. The biotinylated RNAs were incubated with an ultracentrifuged *E*. *coli* extract (S300) at 4°C (to slow down degradation) and then mixed with streptavidin-agarose beads. After an extensive wash, the beads were treated with SDS-containing buffer and the eluted material was separated by urea-PAGE (for RNA, Fig 2A) and SDS-tris-tricine PAGE (for proteins, Fig 2B). The results show clearly that only the U-rich mRNA Ra was co-eluted with a group of small *E*. *coli* proteins in a specific manner under these conditions (Fig 2B, marked by a star). This protein band was excised, analyzed by mass spectrometry, and the main protein content was identified as CspE and CspC, which belong to the CspA family of cold shock proteins [15, 16]. Next, we investigated whether purified 6His-tagged CSPs also preferentially interact with the biotinylated U-rich transcripts Ra and Rc (Fig 2C). The results confirmed that both 6His-CspE and 6His-CspC bind Ra and Rc, significantly better than Rb and Rd.

To characterize the nature of the interaction between U-rich RNAs and CspE further, we studied the effect of magnesium ion (Mg^2+^) on the pull-down outcome. The results show that the specific association of 6His-CspE with the U-rich Ra or Rc was maintained at both the low (2 mM) and high (20 mM) Mg^2+^ concentrations. Clearly however, the complexes were formed less efficiently, but probably more specifically under high Mg^2+^ concentrations (Fig 2D). Importantly, regardless of the Mg^2+^ concentration, both RNAs were fully immobilized and their almost equal release from the beads upon elution was evaluated separately in the various experiments. The effect of Mg^2+^ suggests that the CspE-U-rich RNA association may not be mediated through secondary structural elements in the RNA [17]. These results are not surprising because: (i) the U bias is expected to reduce the formation of secondary structures and (ii) previous studies showed that CspE and CspC recognize very short RNA streches (6–8 bases), which do not form complex secondary structures [18].

Finally, we investigated the interaction of Ra or Rb with CspE or CspC more quantitatively by utilizing a defined system containing purified components. A buffer containing low concentration of biotinylated Ra or Rb was incubated with increasing concentrations of 6His-CspE or 6His-CspC, and immobilized on streptavidin beads. The bound material was eluted by RNase treatment and the amount of released nucleotides was similar in all the samples as assessed by A_260_ measurements (data not shown). The eluated samples were separated by SDS-PAGE and the bands quantified by densitometry after Western blotting with anti CspE antibodies. A representative experiment with 6His-CspC is shown in Fig 2E and 2F and similar results were obtained with 6His-CspE (data not shown). The results of this experiment confirmed our conclusion that CspE and CspC interact specifically with U-rich RNA.

### U-rich RNAs interact specifically with CSPs *in vivo*

**Fig 3:**
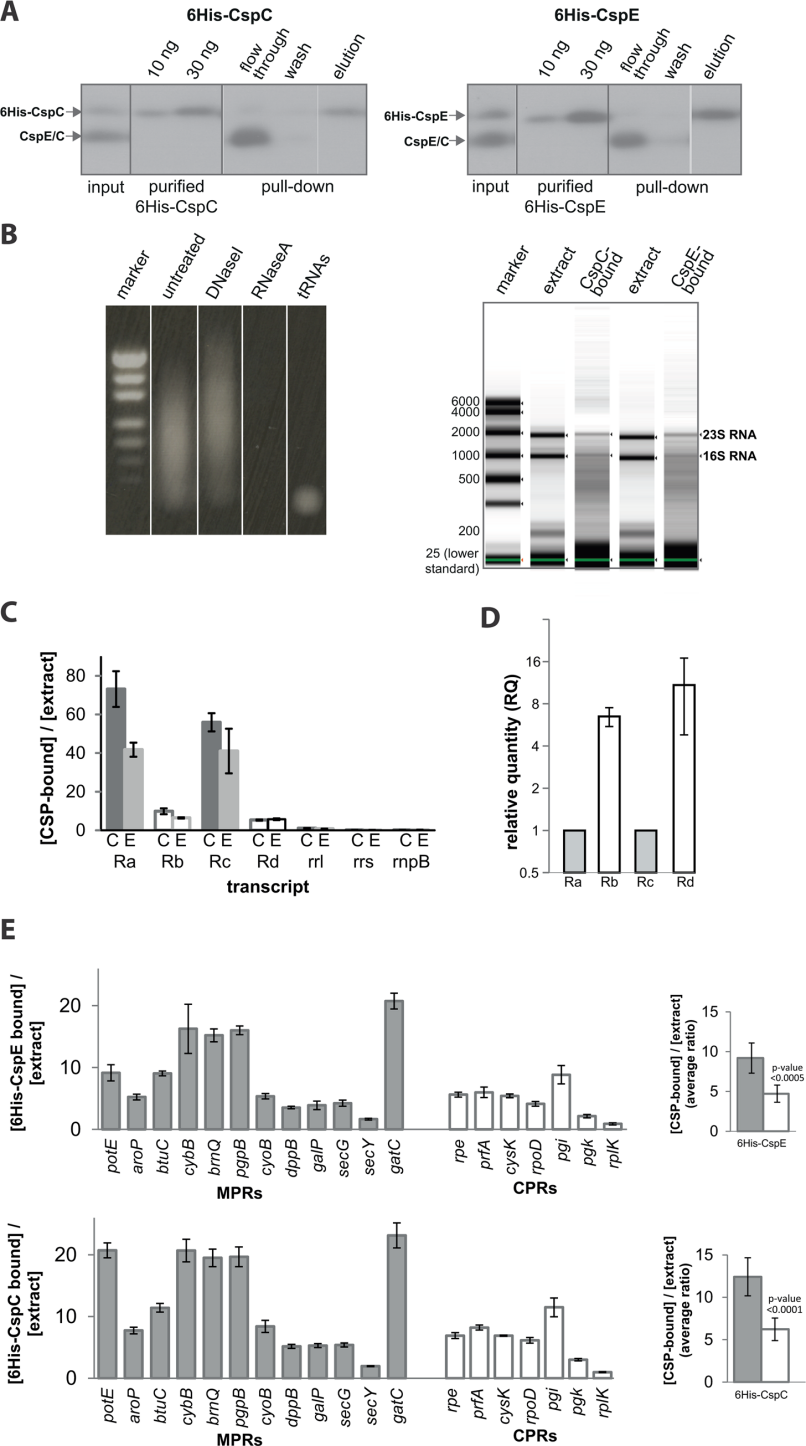
6His-CspE/C pull-down experiments. *E*. *coli* cells co-expressing Ra, or Rb, or Rc, or Rd together with 6His-CspE or 6His-CspC were lysed, and extracts were incubated with Talon resin, for immobilizing 6His-CspE or 6His-CspC and their bound RNAs. **(A)** Samples from the various purification steps were analyzed by Westerm blotting with anti CspE antibodies. **(B)** Left panel, the eluates were treated with DNase or RNase and analyzed by Agarose gel. Right panel, the quality and size of the eluted RNA were analyzed by tapestation. **(C)** The total extract and the CspE/CspC bound RNAs were analyzed by qPCR with the corresponding primers to Ra, Rb, Rc, or Rd. Primers to rrl, rrs, and rnpB were utilized as controls. Values were calculated as 2^extract Ct^ / 2^pull-down Ct^ (see methods). Error bars indicate SEM (n = 3). **(D)** RNA was extracted from disrupted cells and the total steady state amount of R transcripts was measured by qPCR. Endogenous RNAs *ssrA* and *rnpB* were used as controls to assure unbiased results. Since both of the controls were similarly expressed in the various samples, we chose *rnpB* expression as a standard for calculating the relative quantity (RQ) of each R transcripts. The RQ of Ra is defined as = 1. **(E)** 6His-CspE (upper panel) or 6His-CspC (lower panel) were purified from wild type *E*. *coli* extracts with Talon resin. The total extracts and the eluates were analyzed by qPCR with primers to various MPRs and CPRs and analyzed as in **(C)**. Error bars indicate SEM (n = 3). Right panels, an average ratio of [bound]/[total] is shown for each experiment.

The above studies were performed with biotinylated RNA molecules prepared *in vitro*. To determine whether the CspE/C-U-rich RNA interaction also occurs *in vivo* with non-biotinylated RNAs, cells were co-transformed with a plasmid encoding Ra, Rb, Rc, or Rd together with a compatible plasmid encoding 6His-CspE or 6His-CspC. Cell extracts were incubated with Talon beads and the eluted, purified 6His-CspE-RNA or 6His-CspC-RNA complexes were analyzed by Western blotting with anti-CspE antibodies (Fig 3A), total RNA characterization (Fig 3B) and qPCR (Fig 3C and 3E and S2 Fig). Fig 3A shows Western blot analysis of samples from the purification steps, using anti-CspE antibodies. This analysis revealed that the endogenous CspE and CspC proteins (lower bands in Fig 3A) are relatively highly expressed even in cells harboring plasmid-encoded 6His-tagged CSPs. In the case of the 6His-CspE, we analyzed the presence of nucleic acids in the eluate, and the results show clearly that it contains only RNA (Fig 3B, left panel), which was characterized by the tapestation for both eluates (Fig 3B, right panels). The Tapestation analysis also indicates that the 6His-CspC and 6His CspE bound material is highly enriched with RNAs. The input and eluted RNAs were then analyzed by qPCR using R-transcripts or control primers (Fig 3C), and the results show unequivocally that the specific interaction between CSPs and U-rich RNAs (Ra and Rc) is maintained also *in vivo* and is biotinylation-independent. During the subcellular distribution experiments (Fig 1C and 1D), we observed that the expression of U-rich RNAs was specifically low and could have major effects on our pull-down experiments. To test this quantitatively, we measured the amount of the model transcripts at steady state. Fig 3D shows that indeed, the steady-state levels of the U-rich transcripts Ra and Rc were very low compared with those of Rb and Rd. This finding markedly strengthens our conclusion that CspE and CspC interact specifically with the U-rich transcripts, despite their low expression.

**Fig 4:**
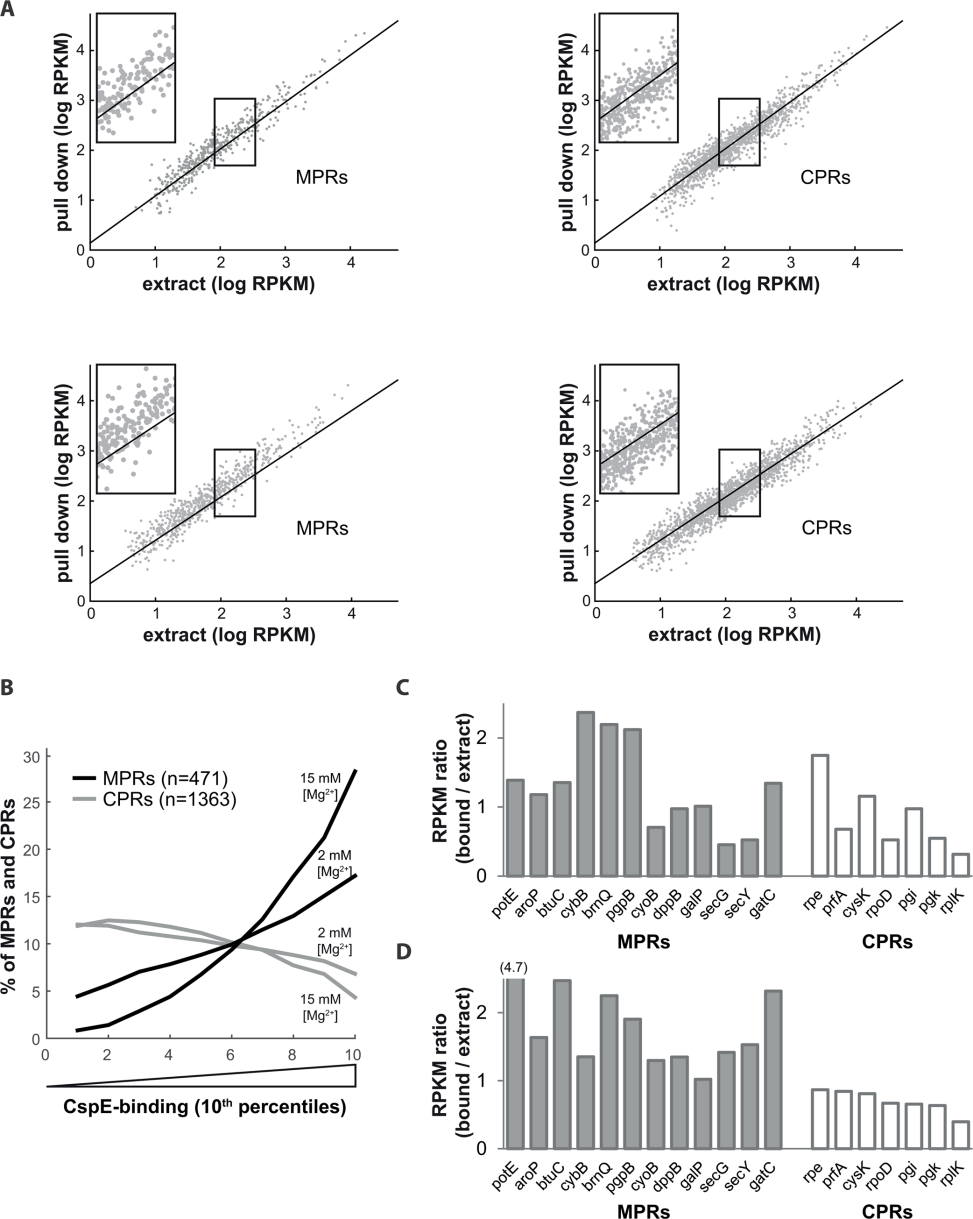
High throughput sequencing of endogenous RNAs that co-purify with 6His-CspE. **(A)** Wild type *E*. *coli* expressing CspE-6His were disrupted in the presence of either 2 mM or 15 mM [Mg^2+^] (top and bottom panels, respectively) and the total cell extracts were subjected to metal affinity chromatography using Talon resin. RNA was prepared from the total cell extracts and the imidazole-eluted material (see Fig 3) and subjected to high throughput sequencing. The amount of CspE-6His bound MPRs (left panels) and CPRs (right panels) is plotted as a function of the amount of the same mRNAs in the total extract. **(B)** CspE-binding of all detected mRNAs was calculated as [RPKM_CspE-bound_ / RPKM_extract_]. The quota of MPRs and CPRs in each 10^th^ percentile along the CspE-association landscape is presented as a moving average plot. **(C)** This panel shows the CspE-binding values in the presence of 2 mM [Mg^2+^] for selected MPRs and CPRs that were similarly analyzed by qPCR (see Fig 3D for comparison). **(D)** An independent experiment that shows the CspE-binding values for selected MPRs and CPRs in the presence of 15 mM [Mg^2+^].

Next, the input and eluted RNAs were analyzed by qPCR using primers specific to various MPRs and CPRs. On average, the results show an interesting pattern of pulled-down MPRs and CPRs (Fig 3E, S2 Fig). Of the few test examples, it is apparent that several MPRs are enrichred in the pulled down material, compared to CPRs, with both 6His-CspE (Fig 3E upper panel) and 6His-CspC (Fig 3E, lower panel). The question why other MPRs, such as SecY are underrepresented is currently being investigated. Preliminary experiments under high [Mg^+2^] conditions revealed an increased specificity of CspE for MPRs, including *secY* (data not shown, and Fig 4), in agreement with the previous studies of the effect of [Mg^+2^] (Fig 2D, 2E and 2F).

### High-throughput analysis of 6His-CspE bound endogenous mRNAs *in vivo*

We have shown above that CspE and CspC interact preferentially with several U-rich model RNAs and endogenous MPRs *in vivo* and *in vitro*. To examine whether these results are representative, we expressed CspE-6His and performed metal-affinity pull-down experiments with total cell extracts under low and high [Mg^+2^] and similar although not identical conditions (see materials and methods). Endogenous RNA was prepared from the total extracts and the pulled-down material, sequenced, and analyzed. The left panels in S3 Fig confirm previously observed associations between CSPs and several CSP-encoding mRNAs [18–21], and in addition it reveals that other *csp*-mRNAs also interact with CspE, more specifically under low concentrations of Mg^+2^. This sampling experiment serves as a positive control in the pull-down assay. Together, the results show that the total extract was enriched with complexes containing CspE-bound MPRs (compare Fig 4A left and right panels). These results are summarized in Fig 4B, which demonstrates that a relatively large population of MPRs are highly CspE-associated, especially under high [Mg^+2^], while CPRs show an opposite trend, as also observed with secretory protein mRNAs (SPRs, S3 Fig, right panels). More specifically, under low [Mg^+2^] conditions, MPRs are significantly enriched within the 30% most CspE-associated mRNAs (p-value = 7.8x10^-16^, 213 of 471 detected MPRs are within that group) and they are relatively depleted from the 30% least CspE-bound genes (only 80 of 471 detected MPRs are within that group). In contradistinction, CPRs are significantly enriched within the 30% least CspE-bound mRNAs (p-value = 3.8x10^-4^, 487 of 1363 detected CPRs are within that group), and are moderately depleted from the 30% most CspE-associated genes (334 of 1363 detected CPRs are within that group). At high [Mg^+2^], the difference between MPRs and CPRs is even more significant (Fig 4B).

Next, in order to compare the qPCR results (Fig 3E) with the RNA-seq results of CspE-bound mRNAs, we selected the same mRNA species that were analyzed by qPCR and presented their relative amount in the high throughput pull-down material compared to their amount in the total extract. Overall, the RNA-seq results also showed preferential binding of the 6His-tagged CspE to MPRs (Fig 4C), and the pattern is similar to that observed in the qPCR results (Fig 3E). As expected, also this analysis revealed that CspE distinguishes between MPRs and CPRs much better under high [Mg^+2^] (Fig 4D).

### Characterization of CspE/C deleted *E*. *coli*

Our results show that U-rich RNAs including MPRs interact specifically with CspE and CspC. An obvious step forward would be to investigate what is the physiological relevance of this interaction. Intriguingly, *E*. *coli* has nine CSP paralogues (CspA-CspI) and it is noteworthy that only several are induced by cold shock (CspA, B, G, I). CspC transcription does not respond to cold shock [22]; however, CspE does, but only slightly [23]. Both CspE and CspC are expressed constitutively at a physiological temperature (37°C) [24]. CspD is a stationary phase protein [25, 26] and CspA is expressed at 37°C, during the early growth stage [27], and transiently under cold shock conditions [28]. The fact that several CSPs are expressed constitutively at a physiological temperature suggests that they play a role that is unrelated to cold adaptation [29–31]. In light of the high number of *csp* genes, obtaining a more defined view of the physiological role of each CSP is not trivial. Having a large battery of 9 paralogues makes it extremely difficult to analyze their role *in vivo* by gene deletion, because they might compensate for one another, and also regulate each other’s expression [e.g., [19]]. Nevertheless, future construction of multiple deletions in all the genes that express under physiological conditions, as was done previously with other combinations [32], is essential for determining the importance of CSPs in recognizing U-rich MPRs and in the biogenesis of membrane proteins.

**Fig 5:**
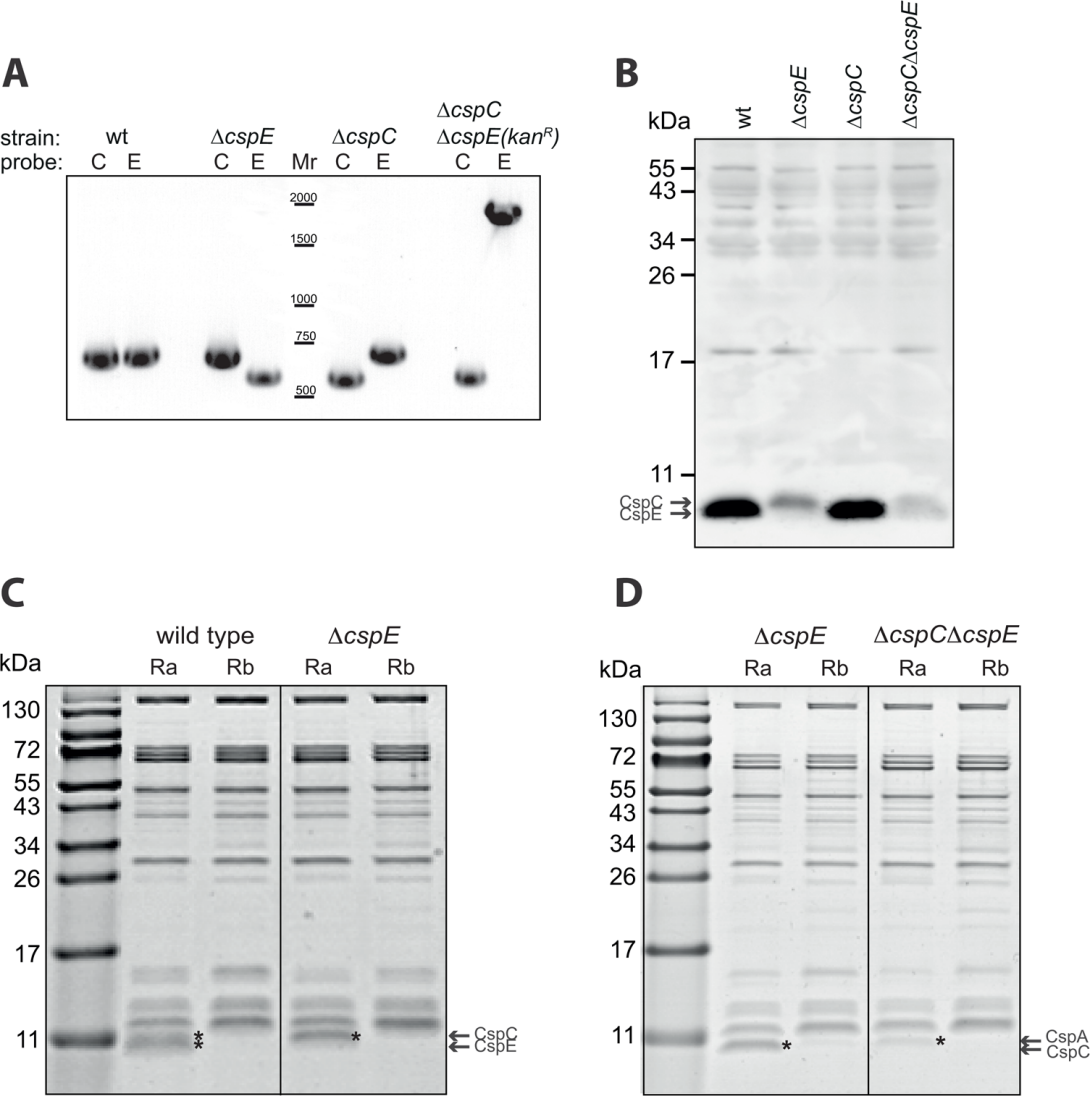
Characterization of CspE-, CspC-, and CspC/E deleted cells. **(A)** PCR analysis of the *cspE* and *cspC* in *E*. *coli* Δ*cspE*, Δ*cspC*, or Δ*cspC*Δ*cspE*::*kan* ^*R*^. **(B)** Western blot analysis with anti-CspE antibodies of total extracts from wild type and the deleted *E*. *coli* strains. **(C) (D)** *In vitro* synthesized biotinylated Ra or Rb were incubated with S300 fractions of wild type *E*. *coli* or *E*. *coli* Δ*cspE*, or *E*. *coli* Δ*cspE* Δ*cspC* in the presence of 2 mM Mg^2+^. The RNA-protein complexes were purified by streptavidin beads, eluted with SDS buffer and separated by tris-tricine SDS-PAGE. The gels were stained with Instant blue.

In an attempt to search for interesting phenotypes in the absence of CspE and/or CspC, we studied *E*. *coli*(Δ*cspE*) *E*. *coli*Δ*cspC*) and a double-deletion *E*. *coli*(Δ*cspE*ΔcspC) strain (see methods). Initially the strains were evaluated both by PCR (Fig 5A) and Western blotting with anti-CspE antibodies (Fig 5B). Growth experiments in rich media showed that all the strains grow as well as wild type *E*. *coli* at 37°C (data not shown). Next, we repeated the co-purification experiments utilizing *in vitro*-synthesized biotinylated Ra and Rb and extracts from the deleted cells. In the Δ*cspE* strain, a small protein was co-purified only with Ra, and the mass spectrometry results revealed that it is CspC, as expected (Fig 5C, left panel, star). Similarly, in the Δ*cspE*Δ*cspC* strain a third small protein was found to poorly interact only with U-rich Ra, and it was identified by mass spectrometry as CspA (Fig 5C, right panel, star).

### Conclusions

The concept of differentiation between subgroups of mRNAs that need to be delivered to specific cellular niches is extremely interesting in bacteria because their mRNAs usually do not contain long 3’ UTRs, which in eukaryotes, serve as guiding elements [33, 34]. Therefore, if bacterial mRNAs are also specifically recognized and localized, they must harbor non-coding information inside coding regions. In our work we evaluated the possibility that cellular factors are specifically involved in the recognition of MPRs that contain long U-rich stretches [14]. CspC and CspE were identified biochemically as specific U-rich RNA-binding proteins and the interactions were verified *in vivo*. Importantly, CspE and CspC are termed cold shock proteins but they express normally under physiological conditions and we show that they interact specifically also with endogenous MPRs. This interaction is most likely primary sequence-dependent because it is strongly affected by magnesium ion concentrations. We hypothesize that these CSPs interact with MPRs during or soon after transcription, and may play a specific role while these mRNAs are present in the cytosol, before targeting the membrane.

## Materials and Methods

### Synthetic sequences and Plasmids

Synthetic genes encoding Ra, Rb, Rc, and Rd (S1 Fig) were produced by GenScript, and delivered as non-directional ligated inserts within pUC57 vector linearized by EcoRV digestion. These genes were consequently transferred to expression plasmids by standard PCR amplification (primers are described in S1 Table), followed by digestion with XbaI, phosphorylation, and ligation into the low copy number plasmid pZS*12-luc [35], digested with XbaI and EcoRI and treated by Klenow (plasmids are described in S2 Table). For insertion of Ra-d into pT7-5 [36] under the T7 promoter, Ra and Rb were excised from the Genscript plasmids with BamHI and HindIII, Rc was digested with SacI and HindIII, and Rd was amplified by PCR using pZS*12-Rd and digested with SacI and HindIII. Then, Ra-d were ligated into pT7-5, digested with the corresponding enzymes. Throughout the study we used the following plasmids for 6His-tagged CspE/C expression: pET28a-*6His-cspE/C* (T7 promoter, inducible in *E*. *coli* BL21), pIE1-*cspE-6His* and pDBH2-6His-*cspE/C* (both are arabinose inducible, and pDBH2-*6His-cspE/C* is compatible with pZS*12-Ra-d plasmids) (S2 Table). The *cspE* and *cspC* genes were amplified by colony PCR using *E*. *coli* BW25113 as template (all strains are described in S3 Table) and ligated into pET28a (digested by NdeI and SacI). In-order to create pDBH2-6His-*cspE/C*, the *amp* ^R^ gene was removed from pT7-5(*amp*)(*kan*)(*araP*)-*ffh-6His* [37] by PCR and self-ligation to create pDBH1. Next, 6His-*cspE/C were* amplified from pET28a-6His-*cspE/C*, and inserted by linear amplification (RF cloning) instead of the *ffh*-*6His* gene. For construction of pIE1-*cspE-6His*, *araP* was amplified by PCR using pIY212, [37] as a template and ligated into pET28a-*6His-cspE* (digested by XbaI and NdeI) to create pET(*araP*)-*6His-cspE*. Next, a PCR product containing *araP-cspE* from pET(*araP*)-*cspE* and a PCR product containing OriR, *amp* ^R^, and the 6His-tag from pIY212, were ligated (using SalI and BamHI).

### General cell growth and expression conditions

Unless mentioned otherwise, colonies were inoculated for overnight growth at 37°C in LB medium, supplemented with ampicillin (100 μg/mL), kanamycin (30 μg/mL), chloramphenicol (10 μg/mL), or tetracycline (6 μg/mL), when necessary. Overnight cultures were diluted to an optical density (A_600_) of ~0.05 and grown in similar media and conditions. Expression of R-transcripts from pZS*12-Ra-d was induced at mid logarithmic phase (A_600_ = 0.5) using 0.5 mM IPTG for 30 min. For analysis of the *in-vivo* interaction of CspC or CspE with Ra-Rd, the R transcripts were expressed from pZS*12-Ra-d and 6His-CspE/C were expressed simultaneously from pDBH2-6His*-cspE/C*. For analysis of the *in vivo* interaction of CspE with endogenous mRNAs by RNA-seq, CspE-6His was expressed from pIE1-*cspE-6His*. Expression was induced with 0.1% arabinose.

### *In vitro* synthesis of Ra-Rd RNA with or without biotin labeling

RNAs were produced by *in vitro* transcription using linearized pT7-5 plasmid encoding each of the R transcripts and components of T7-MegaScript kit (Ambion). A typical reaction mixture (50 μL) contained 1–2 μg plasmid linearized by HindIII digestion, enzymes and 7.5 mM of each of the kit-provided nucleotides tri-phosphates. In order to produce biotin-modified RNA, 1.5 mM of the CTP (out of 7.5 mM) was replaced by 1.5 mM biotin-11-CTP (Roche Diagnostics). After 4 h incubation at 37°C, the mixture was cooled down on ice and RNA was precipitated by LiCI (final concentration 2.5 M). After incubation for 1 h on ice and centrifugation for 15 min at 10,000g, pellet was washed with ice-cold 2.5 M LiCI, 75% Ethanol and solubilized in 100 μL 0.2 M Na-acetate, pH 5.5. RNA was precipitated with 2.5 volume of ethanol at -80°C over night. The RNA pellet was collected by centrifugation (20 min at 10,000g, 4°C), washed with 75% ethanol and solubilized in 100–150 **μ**L H_2_O. RNA preparations were analyzed by A_260_ measurements (NanoDrop) and by electrophoresis on 2% agarose gel in TAE buffer (1 mM EDTA and 75 mM Tris-HAc, pH 8.0) and by 6% PAGE in TAE containing 8 M urea, and stored at -80^0^.

### Expression and purification of 6His-CspE and 6His CspC

*E*. *coli* BL21(DE3)p*LysE* harboring pET28a-6His-*CspE* was grown in LB medium, supplemented with 30 μg/mL kanamycin and 10 μg/mL chloramphenicol at 37°C and induced with 0.5 mM IPTG at the exponential growth phase (A_600_ = 0.5) for 2.5 h. Cells were harvested by centrifugation for 25 min (7,000g, 4°C). For cell extract preparations, cell pellets from 0.5 L cultures were suspended in 10 mL buffer MNT (2 mM MgCI_2_, 0.25 M NaCl, 50 mM Tris/HCI, pH 8.0 and 0.2 mM β-mercapto-ethanol). After addition of 20 units of RNase-free DNase (Promega) and 1 mM pefabloc, cells were disrupted by 3 rounds of sonication (15 sec each) on ice using Sonics (Vibra-cell). Cell debris and unbroken cells were removed by centrifugation at 7,000g for 30 min at 4°C. The 6His-tagged CspE was purified from S160 fraction after ultracentrifugation of a cell extract for 1.5 h at 160,000g. Talon metal affinity resin (Clontech) was used for purification, which was performed in NT buffer (MNT buffer without MgCI_2_ and β-mercaptoethanol) supplemented with 20 mM imidazole (pH 8.0) at the washing step. The protein was eluted with the same buffer containing 250 mM imidazole.

In order to remove RNA that co-purified with 6His-tagged CspE, a second round of metal affinity purification was performed under denaturing conditions. The originally eluted material was dialyzed against buffer GT (10% glycerol, 50 mM Tris/CI, pH 7.5 and 0.2 mM β-mercaptoethanol) to remove the imidazole and then incubated in 8 M Urea-NT solution for 1 h at RT and 0.5 h on ice to dissociate bound RNA. The second round of metal affinity purification was performed in NTU solution (250 mM NaCI, 50 mM Tris/CI, pH 8.0 and 6 M Urea). Washing of the Talon resin after loading of Urea-denaturated 6His-tagged CspE and protein elution were performed in NTU supplemented by 10 mM or 200 mM imidazol, respectively. RNA-free 6His-tagged CspE was then re-natured by extensive dialysis against GT buffer. The concentration of purified CspE was estimated by Bradford assay with BSA as a standard. 6His-CspC was overexpressed in *E*. *coli* BL21(DE3)/pLys harboring pET28a-6His-*cspC* and purified as described for 6His-CspE.

### Preparation of *E*. *coli* cell extracts and S300

*E*. *coli* extracts were produced by sonication as described previously [38], with minor modifications. Cell pellets were suspended in ice-cold 5% sucrose solution in buffer MKNH (2–20 mM MgCl_2_, as indicated, 20 mM KCI, 100 mM NH_4_CI, 20 mM HEPES, pH 7.5, and 0.2 mM DTT) or MNT buffer (see above) to cell density of 40–45 A_600_. After addition of 5 U/mL RQ1 DNase (Promega) and 1 mM pefabloc, cells were sonicated 3x10 seconds and cell debris was removed by centrifugation (15 min, 10,000g, 4°C). For preparation of S300 fractions, cell extracts were ultracentrifuged at 300,000g (1.5 h, 4°C) and the supernatant was collected.

### Sucrose gradient ultracentrifugation

Cell extracts were prepared as described above in buffer MKNH containing 15 mM MgCl_2_, with addition of 0.1 mM EDTA and 250 U/mL RNasIn plus (Promega). 500 μL cell extract was loaded on top of a 12 mL 7–22% linear sucrose gradient prepared in the same buffer, and ultracentrifuged (260,000g, SW41 rotor, Beckman for 3 hours at 4°C). 600 μL fractions were collected (top to bottom) and the pellet was resuspended in 600 μL of 7% sucrose solution. A_260_ was measured for each fraction using a NanoDrop spectrophotometer.

### His-tagged CspE/C pull-down assays

*Cell pellets of E*. *coli* BW25113 harboring pIE1–*cspE-6His or* (pDBH2-*6His-cspE/ or C* + pZS*12-*Ra*, *Rb*, *Rc*, *Rd*) were suspended (to A_600_~40) in buffer with low Mg^+2^ concentration (5% Sucrose, 50 mM Tris pH 8, 150 mM NaCl, 2 mM MgCl_2_, 0.2 mM β-mercaptoethanol) or high Mg^+2^ (20 mM HEPES, pH 7.5, 20 mM KCl, 100 mM NH_4_Cl, 15 mM MgCl_2_, 0.2 mM β-mercaptoethanol) and cell extracts were prepared as above in the presence of pefabloc, RQ1 DNase, and RNasIn plus. The cell extracts were supplemented with 5 mM imidazole and mixed with pre-equilibrated Talon beads (0.05 mL per 1 mL extract). The beads were incubated with rotation for 30 min at 4°C, transferred to a column, washed once with 0.5 mL of 50 mM Tris pH 8, 150 mM NaCl, 5 mM imidazole and 2 more times with 0.5 mL of the same buffer containing 20 mM imidazole. His-tagged CspE/C and bound RNA were eluted with 250 mM Imidazole, 300 mM NaCl, 50 mM Tris pH 8.

### Isolation of biotinylated RNA-proteins complexes

Isolation of complexes between biotinylated RNAs and S300 proteins was performed at 4°C, at conditions where Ra-Rd RNAs were stable during long incubation in the presence of S300 extracts (5–7 h). A mixture of S300 (0.7 mg total protein) with 25 μg of the biotin-labeled or non-labeled *in vitro* synthesized R transcript was incubated for 1.5 h at 4°C in 0.35 mL buffer Ai (buffer MKNH supplemented with 0.01% Igepal CA-630) supplemented with 110 units of RNasin plus (Promega). Next, the mixture was incubated with 50 μL of pre-equilibrated streptavidin agarose beads (Novagen) for 1 h at 4°C in the rotator (Intelli-mixer). The beads were collected by centrifugation for 1.5 min at 7,000g and washed 4 times with 0.6 mL and once with 0.25 mL of ice-cold Ai. The protein-RNA complexes were eluted from the beads with 80 μL of SDS-urea solution (2% SDS, 8 M urea, 10 mM EDTA and 100 mM Tris-HCI, pH 8.0). The eluted RNA samples (15 μL) were separated by 6% PAGE in TAE containing 8 M urea. RNA gels were stained with ethidium bromide. The co-eluted protein samples were incubated for 25 min at 37°C in the presence of DTT (30 mM) and b-mercapto-ethanol (100 mM). 50 or 5 μL of samples were separated by Tris-Tricine SDS-PAGE (separation gel with 14% T, 3% C) and stained by Instant-Blue or subjected to Western blot analysis, respectively.

In order to isolate RNA complexes with purified 6His-tagged CspE or CspC *in vitro*, 20 μg purified protein were incubated with 18 μg of biotinylated R transcript and 30 μg *E*. *coli* total tRNA (Boehringer Mannheim) in 250 μL Ai supplemented with 75 units of RNAsin for 1 h at 4°C. Isolation of complexes and their analyses by electrophoresis were performed as described above. The amount of CspE/C bound to the eluted RNA in the different samples was quantified by densitometry.

For studying concentration dependent CspE/C binding to biotinylated RNAs, a mixture containing 6 μg biotinylated RNA (0.17 μM), 30 μg *E*. *coli* total tRNA, 10 μg BSA, 75 units of RNasin and increasing amounts of purified 6His-CspE or 6His-CspC (0.43–5.4 μg or 0.17–2.2 μM, respectively) were incubated in 250 μL Ai buffer with 15 mM MgCI_2_ for 1.5 h at 4^0^ C. All components except of biotinylated RNA were included also in a control mixture. Isolation of the complexes was performed with 30 μL Streptavidin beads as described above. Analysis of unbound material by RNA-PAGE showed that the biotinylated RNA was fully immobilized. After intensive wash of the beads the RNA-protein complexes were eluted by 50 μL SDS-B solution (1.2% SDS, 10 mM EDTA, 100 mM Tris-HCI pH 8.0, 10% glycerol and 1 mM DTT). A_260_ measurment (NanoDrop) confirmed that the eluted RNA amounts were the same in the various samples (15–20% of the input). In order to elute RNA more quantitatively, RNaseA treatment was performed. The beads were suspended in 60 μL of GTE solution (15% glycerol, 0.012% Igepal, 150 mM NaCI, 2 mM EDTA, 100 mM Tris/CI, pH 8.0 and 1 mM DTT). RNaseA (50 μg/mL) was added and the samples were incubated for 1 h at room temperature. Then, SDS was added (1.2%) and the samples were centrifuged for 1.5 min at 7,000g at room temperature. A_260_ measurment (NanoDrop) showed that the RNaseA treatment released 55–65% of the biotinylated RNA input. The first elution (with no RNase treatment) was combined with the second one (after RNase treatment) and the total RNA concentration was measured. The eluted CspE/C in samples containing equal amounts of eluted RNA, was analyzed by Tris-Tricine SDS-PAGE and Western blotting with anti-CspE and quantified by densitometry.

### Construction of *csp*-deleted strains

*E*. *coli* BW25113Δ*cspE*:*kan* and Δ*cspC*:*kan* were utilized as templates [39]. Kanamycin resistance was removed from BW25113Δ*cspC*:*kan* by transformation with pCP20 [40]. *E*. *coli* BW25113Δ*cspE*:*kan*Δ*cspC* was constructed by P1 transduction using BW25113Δ*cspC* as acceptor and P1 lysates of BW25113Δ*cspE*:*kan*. Positive transductants were selected on LB-agar plates containing 30 μg/mL kanamycin and verified by PCR, sequencing, and Western blotting.

### Western blotting

SDS-PAGE and Western blotting were performed as described [38]. Rabbit anti-CspE polyclonal antibodies were prepared in the course of this study with purified 6His-CspE by the Antibody Unit of the Weizmann Institute. Goat anti-rabbit antibodies conjugated to horseradish peroxidase were used as secondary antibodies (Jackson Immunoresearch).

### RNA extraction and quantitative PCR (qPCR)

Typically, RNA was extracted from 400 μL-samples with 400 μL of water-saturated Biophenol (Tris-buffered Phenol:Chloroform:Iso Amyl Alcohol 25:24:1). Mixtures were vortexed, incubated 10 min at room temperature and centrifuged (10 min, 12,000g at 4°C). 150 μL from the top aqueous phase were mixed with 150 μL of water-saturated chloroform. Mixtures were vortexed and centrifuged (10 min, 12,000g) and 70 μL of the top aqueous phase were mixed with 7.7 μL of 2 M sodium-acetate pH 5.3 and 196 μL of cold ethanol. Mixtures were vortexed, stored overnight at -80C°, and then centrifuged (15 min, 14,000g, at 4°C). Supernatants were removed and the pellets were washed twice with 75% ethanol. The isolated RNA was dissolved in DEPC-treated water (15–50 μL) and its concentration was measured by NanoDrop. DNaseI treatment and removal was performed using DNA-free kit (AM1906, Ambion). cDNA was synthesized using a high capacity cDNA reverse transcription kit (Applied Biosystems). qPCR was performed using power sybr green (Applied Biosystems), and an ABI 7300 or ViiA7 machine (qPCR primers are listed in S4 Table). Relative quantities were assessed using RnpB, SsrA and occasionally also 16S or 23S rRNA as endogenous controls. All experiments included at least two endogenous controls. Ratios of fraction or pull-down to extract concentrations were calculated for all genes as 2^input Ct^ / 2^fraction Ct^. The input is the extract that was either fractionated or used for pull down. Ct is cycle of threshold, which was 0.2 for all genes. PCR efficiency of all primers was verified by standard curves with -3 ≥slope≥ -3.6, R^2^>0.995.

### RNA-seq

Libraries were prepared as previously described [41]. Essentially, triplicate samples of rDNaseI treated RNA (1 μg) were fragmented at 70°C for 5 min (extract RNA) or 3 min (CspE-bound RNA), using RNA fragmentation kit (Ambion, AM8740). Fragmented RNA was purified using AMPure magnetic beads (Agencourt A63881) at a 2.2/1 ratio and reverse transcribed at a final volume of 12 μL. Second strand cDNA synthesis was performed by addition of the following: 3 μL NEB2 10X buffer, 1.2 μL dNTPs 10 mM, 1.2 μL dATP 10 mM, 0.8 μL *E*. *coli* DNA polymerase (NEB, M0209), 1.6 μL RNaseH (NEB, M0297), 0.4 μL T4 DNA ligase (NEB, M0202). Final volume was adjusted to 30 μL using nuclease free water and the reaction was incubated at 16°C for 2.5 h. Double strand cDNA was purified using magnetic beads, and undergone A-addition using KLENOW exo^-^ (NEB, M0212) in NEB buffer 2 supplemented by 167 μM dATP for 30 minutes at 37°C. Reaction product was purified using magnetic beads and ligated to adapters carrying the Illumina sequences using Quick ligation kit (NEB, M2200) as described. The resulting libraries were amplified with 14 cycles of PCR using the PFUultraII fusion (Agilent). Libraries were sequenced by the INCPM center (Weizmann Institute of Science), on the Hiseq2500 (50 bases, single read). The sequencing data were submitted to the National Center for Biotechnology Information Sequence Read Archive under Accession No. SRP060022.

### Data analysis

Reads were mapped to the corresponding reference genome (NC_000913) using in-house scripts. Data analysis was performed using in-house Matlab scripts. Triplicates were pooled after validation for consistence. rRNAs, tRNAs and additional non-coding loci-mapped reads and poorly-detected mRNA reads were removed. Reads Per Kilobase per Million mapped reads (RPKMs) were calculated for 2731 protein-encoding mRNAs that were sufficiently detected in both experiments. MPRs were defined according to Uniport location SL-9909 (multi-pass membrane proteins), of which several annotated “outer membrane” protein coding mRNAs (SL-0040) were discarded. Cytoplasmic proteins were defined according to the PSORT database [42, 43] and modified by the removal of the 9 *csp* homologous genes. Secretory proteins were also defined by the PSORT database by pooling periplasmic, outer membrane and extracellular proteins. Unless mentioned otherwise, calculation of Bonferroni-corrected p-values for RNA-seq data analysis was performed using the DAVID functional annotation tool [44, 45]. The RNA-seq data that we analyzed here is shown in S5 Table.

## Supporting Information

S1 Fig DNA sequences encoding RNAs Ra-Rd. (PDF)

S2 Fig Relative quantity of mRNAs in cells expressing 6His-CspE or 6His-CspC. (PDF)

S3 Fig Interaction of CSP-encoding mRNAs and mRNAs encoding secretory proteins with CspE *in vivo*). (PDF)

S1 Table Cloning primers. (PDF)

S2 Table Plasmids. (PDF)

S3 Table *E*. *coli* strains. (PDF)

S4 Table qPCR primers. (PDF)

S5 Table RNA-seq data. (XLSX)
