# Supplementary material for: Model Uracil-Rich RNAs and Membrane Protein mRNAs Interact Specifically with Cold Shock Proteins in Escherichia coli
Source: PLoS One. 2015 Jul 30;10(7):e0134413. doi: 10.1371/journal.pone.0134413 (PMC4520561; doi:10.1371/journal.pone.0134413)
Supplement: S1 Fig — (PDF) [file pone.0134413.s001.pdf]

Ra

**TGATACTATTTGAAAAACACATAAAACTTTTGGGTATTCGGTTTATTCTTTTTCTTTTACTTTTTTATCTG
AGGAGCCTACTTCCCGTTTTTCCCGATTTGGCTACTGAACATCAACCATATCAGCTAAAAAAGTCATACGG
GTATTATTTTTGCCGCTATTTCTCTGTTCTCGCTATTATTCCAACCGCTGTTTGGTCTGCTTTCTGACAAA
CTCGGGCTGCGCAAATAATACCTGCTGTCGATTATTACCGGCTGATTAGTCTGATTTGCGCCGTTCTTTAT
TTTTATCTTCGGGCCACTGTTACAATACAACATTTTATAAGTAGGATCGATTGTTGGTCGTATTTATCTAG
GCTTTTGTTTTAACGCCGTCGCGCCAGCAGTAGAGGCATTTATTGAGAAAGTCAGCCGT**

Rb

**ATTGAAAGTAGTCTGCTGCTGCTGAACGGCAAGCCGTTGCTGATTCGAGGCGTTAACCGTCACGAGCATCA
TCCTCTGCTGAGTCAGGTCTGAGGATAGCAGACGTAGGTCCAGGATATCCTGCTGGATAAGCAGAACAACT
TTAACGCCGTCCGCTGTTCGCATTATCCGAACCATCCGCTGTCGTACACGCTGTCCGACCGCTACGGCCTG
TTGCTGGTCGGATAAGCCAATATTGAAACCCACGGCTGAGTCCCAGATAATCGTCTGACCGGTAATCCGCG
CTGGCTACCGGCGGTAAGCGAACGCGTAACGCGAGTAGTCCAGCGCGATCGTAATCACCCGAGTCTGATCA
TCTGGTCGCTGGGGAGATAATCAGGCCACGGCGCTAATCACGACGCGCTGTATCGCTGG**

Rc

**GCTACGCGATCGCGgtaGGGTAAGTTCAAACCCTGaATCCTGATCGGTACGTTGGCAAACTCTGTAATCTT
ATTTCTCCTCTTTAGTcCGCATCTGTTTGAAGGTACTACTtAGATTGTCTTTGTTTGCGTcACCTACATCC
TCTGGGGCgtaACTTACACCATTgtaGATATTCCCTTCTGGTCGCTGGTTCCAACCATCACGCTCGATAAA
CGTcAGCGCGAAtAACTGGTTCCTTATCCGCGTTTTTTTGCCAGTCTGGCAGGCTTTGTTACGGCAGGTcT
GACGCTACCATTTGTTAATTgtaTCGGCGGTcGCGATCGGtaATTTGGCTTTCAGgtaTTCACTCTGGTAC
TGATCGCCTTTTTTATTGTTTCAACCATCATCACTCTGCGCAgatTGCgatAAGTCTTT**

Rd

**ACCATTGCCGATACGTaGGGGCCGGGCGGTATTgtaCGCGCGCTACGTACCATTCCGCATCTGTaGCAAAT
TTGCGAGGACgtaACGGAAGTCTGCCCCGgtaCCACCgtaCTCAACTgtaTTAACCCAgtaGCGgtaAATA
CCTGGGCGgtaTagaCCCGCTATCCGCATATCAAACAGGTCGGGCTGTaaCATTCGGTcCAGGGAACGGCG
GAAGAGTTGGCGCGTcACCTCAATATCGACCCAGCTACGCTGCGTTACCGTTGCGCAGGTATCAACCATtg
aGCGTaTTACCTGGAGCTGGAGCGCAAAACCGCCGACGGCAGTTgatTGAATCTCTACCCGGAACTGCTGG
CGGCTTgtaAAGCAGGGCAGGCACCGAAGCCGAATATTCgtaGCAATACTCGCTGCCAG**

Figure S1. DNA sequences encoding RNAs Ra-Rd. Ra-Rd are 414 bp-long DNA sequences, designed to express transcripts that are devoid of start codons, and contain internal stop codons in all reading frames. These transcripts were coined by a capital "R" followed by a lowercase letter: ‘a’ or ‘c’ for sequences that mimic MPRs, and ‘b’ or ‘d’ for sequences that mimic CPRs. *E. coli* genes encoding the IMP lactose permease – LacY (N-terminal 138 amino acids that include 4 TMDs), and the soluble protein, β-galactosidase – LacZ (amino acids 336-473) were used as templates for Ra and Rb respectively. The genes encoding the IMP melibiose permease – MelB (amino acids 64-201 that include 4 TMDs), and the soluble protein, melibiase – MelA (amino acids 110-285) were used as templates for Rc and Rd respectively.
