# Supplementary material for: Model Uracil-Rich RNAs and Membrane Protein mRNAs Interact Specifically with Cold Shock Proteins in Escherichia coli
Source: PLoS One. 2015 Jul 30;10(7):e0134413. doi: 10.1371/journal.pone.0134413 (PMC4520561; doi:10.1371/journal.pone.0134413)
Supplement: S2 Fig — (PDF) [file pone.0134413.s002.pdf]

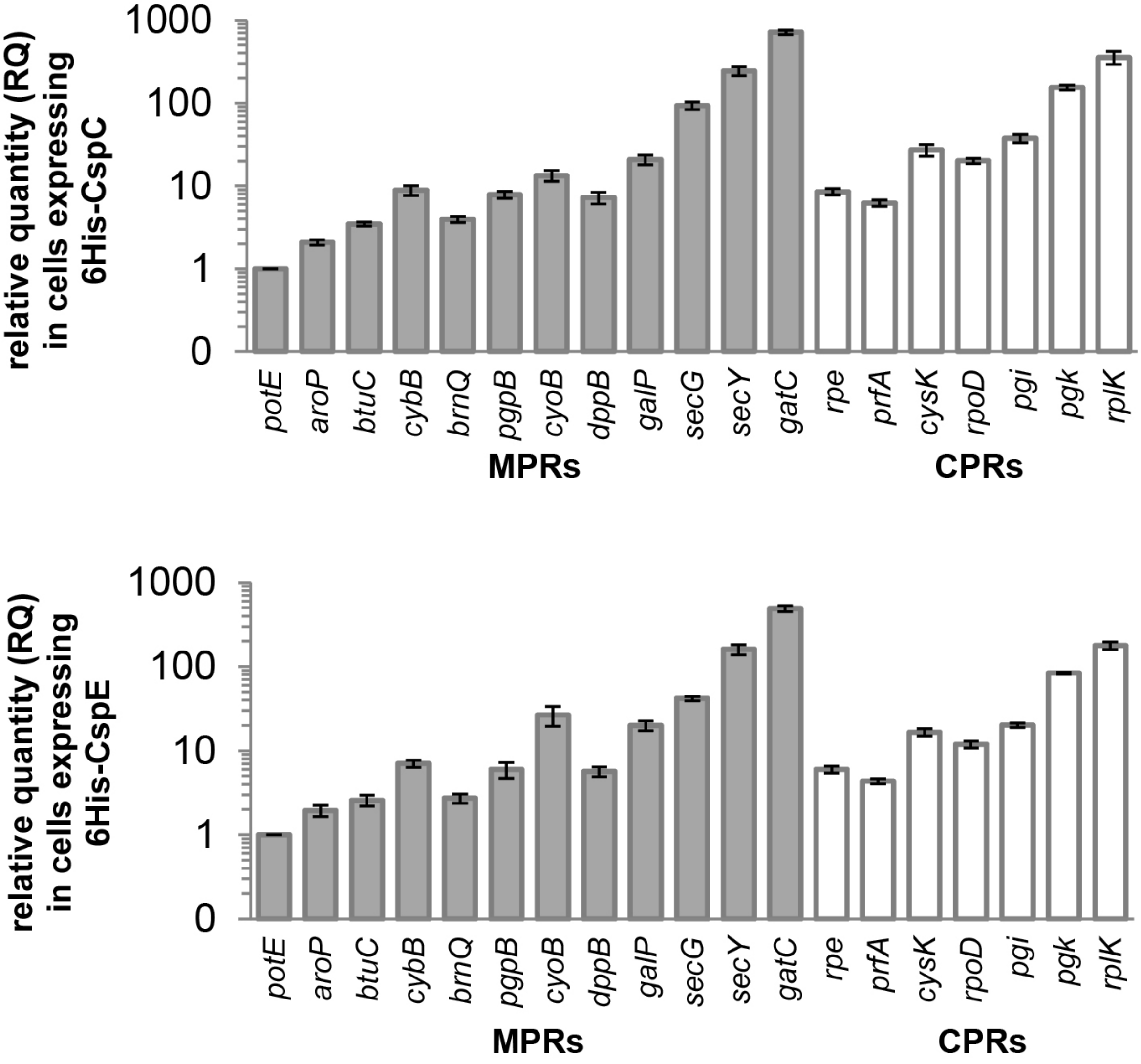

Figure S2. Relative quantity of mRNAs in cells expressing 6His-CspE or 6His-CspC. Wild type *E. coli* expressing 6His-CspC (upper panel) or 6His-CspE (lower panel) were disrupted and the total cell extract was subjected to qPCR analysis with primers to various MPRs and CPRs. Error bars indicate SEM (n=3).
