# Supplementary material for: Model Uracil-Rich RNAs and Membrane Protein mRNAs Interact Specifically with Cold Shock Proteins in Escherichia coli
Source: PLoS One. 2015 Jul 30;10(7):e0134413. doi: 10.1371/journal.pone.0134413 (PMC4520561; doi:10.1371/journal.pone.0134413)
Supplement: S3 Fig — (PDF) [file pone.0134413.s003.pdf]

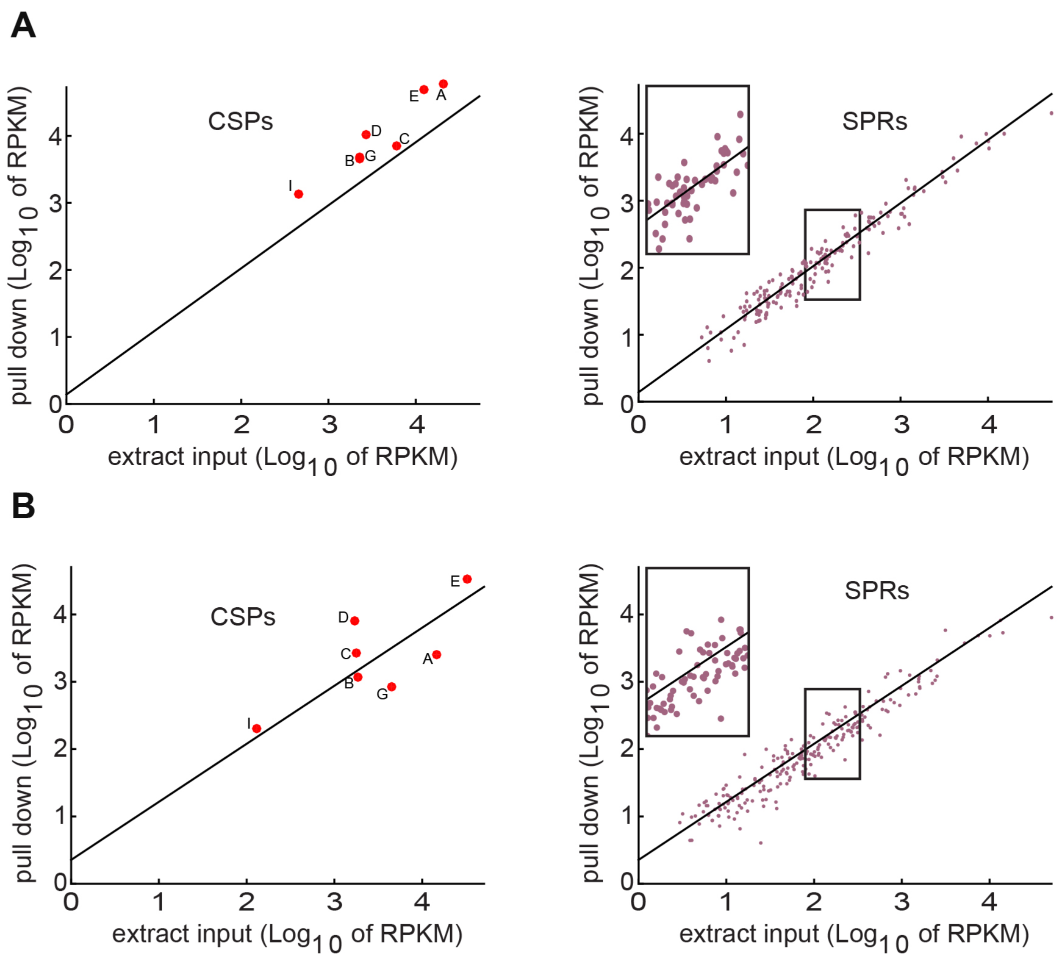

Figure S3. Interaction of CSP-encoding mRNAs and mRNAs encoding secretory proteins with CspE *in vivo*. Wild type *E. coli* expressing CspE-6His were disrupted in the presence of 2 mM (**A**) or 15 mM (**B**) [Mg^{2+}] and the total cell extract was subjected to pull down with Talon beads. RNA was prepared from the total cell extract and the imidazole-eluted material (see Fig. 4) and analyzed by high throughput sequencing. The amount (in RPKM) of mRNAs encoding CSPs (left panels) or secretory protein (SPRs) (right panel) in the CspE-6His bound fraction is plotted as a function of their amount in the total extract. A letter next to each dot in the left panels indicates the represented CSP (A,B,C,D,E,G,I). A linear regression line of all detected genes is presented for reference.
