# Supplementary material for: Model Uracil-Rich RNAs and Membrane Protein mRNAs Interact Specifically with Cold Shock Proteins in Escherichia coli
Source: PLoS One. 2015 Jul 30;10(7):e0134413. doi: 10.1371/journal.pone.0134413 (PMC4520561; doi:10.1371/journal.pone.0134413)
Supplement: S1 Table — (PDF) [file pone.0134413.s004.pdf]

Table S1. Cloning primers.

Name	Sequence (5' NNN…NNN 3')	Use
Ra to pZS*12_fwd	TGATACTATTTGAAAAACAC	Transfer Ra-Rd from pUC57 to pZS*12
Rb to pZS*12_fwd	ATTGAAAGTAGTCTGC	
Rc to pZS*12_fwd	GCTACGCGATCGCGGTAGGGTAAG	
Rd to pZS*12_fwd	ACCATTGCCGATAC	
Ra,b,d to pZS*12_rev	ATGTGCTGCAAGGC	
Rc to pZS*12_rev	GCTCGTATGTTGTGTGGAATTG	
colony cspE_fwd	ACATGCATATGTCTAAGATTAAAGGTAACGT	Colony PCR of cspE into pET28a
colony cspE rev	AAGAGCTCTTACAGAGCGATTACGTTTGCA	
colony cspC_fwd	TGTAGCATATGGCAAAGATTAAAGGTCAGG	Colony PCR of cspC into pET28a
colony cspC rev	ACTTGAGCTCTCAGATAGCTGTTACGTT	
removeAmp_fwd	GTTGAGATCCAGTTCGATGTAAC	Removal of Amp^R from pT7-5(amp)(kan)(araP)(*ffh-6H*)
removeAmp_rev	ACTCTAGCTTCCCGGCAAC	
6H-CspE/C_MegaPrimer_fwd	TGTTTTTCTGGATGGAGTAAGACCCTCGAGATGGGCAGCAGCCATCATCAT	RF insertion of 6H-CspE/C instead of *ffh-6H* in pDBH1
6H-CspE_MegaPrimer_rev	GTTTGACAGCTTATCATCGATAAGCTTTTACAGAGCGATTACGTTTGCAGCA	
6H-CspC_MegaPrimer_rev	GTTTGACAGCTTATCATCGATAAGCTTTCAGATAGCTGTTACGTTAACAGCT	RF insertion of 6H-CspC instead of *ffh-6H* in pDBH1
pZS-Rd_fwd	AAAGAGCTCGAATTACCATTGCCGATACG	Transfer Rd from pZS*12-Rd into pT7-5
pZS-Rd_rev	GCCCAGTCTTTCGACTGAGC	
AraP-1S	CAAATCTAGACCATTCGCTTCAATTGGCG	Amplify AraP from pIY212
AraP-2as	CCACATATGGGTCTTACTCCATCCAGA	
pET-1	GGCTGGAATGTCCACATTG	Amplify araP-csp from pET(araP)-*cspE*
pETas-2	CTTGTCGACGGAGCTCTTTCAGAGCG	
His6 plus	ATAGTCGACATCATCATCATCATCATCAT	Amplify 6H-AmpR-OriR from pIY212
piYas-2	GGTCTTACTCCATCCAGAA	
