# Supplementary material for: Model Uracil-Rich RNAs and Membrane Protein mRNAs Interact Specifically with Cold Shock Proteins in Escherichia coli
Source: PLoS One. 2015 Jul 30;10(7):e0134413. doi: 10.1371/journal.pone.0134413 (PMC4520561; doi:10.1371/journal.pone.0134413)
Supplement: S2 Table — (PDF) [file pone.0134413.s005.pdf]

Table S2. Plasmids.

Name	Source	Use / Properties
pCP20	[1]	Removal of Kan^R
pUC57_Ra-d	Genscript	Source of synthetic R sequences
pZS*12-luc	[2]	Low copy number vector
pZS*12-Ra/b/c/d	This study	Expression of R transcripts
pT7-5	[3]	Vector for in-vitro transcription
pT7-Ra-d	This study	In-vitro transcription of Ra-d
pET28a	Novagen	T7 promotor for protein overexpression
pET28a(*6H-cspE/C*)	This study	Expression and purification of 6H-CspE or 6His-CspC
pT7-5(amp)(kan)(araP)(*ffh-6H*)	[4]	
pDBH1(pT7-5(kan)(araP)-*ffh-6H*)	This study	For cloning pDBH2-*6H-cspE*
pDBH2-6H-cspE(pT7-5(kan)(araP)-*6H-cspE/C*)	This study	Compatible with pZS*12_Ra-Rd.
pIY212(pT7-5(amp)(araP)(*ffh-6H*))	[5]	Source for constructing pIE1-*cspE-6H*
pET(araP)-*cspE*	This study	Intermediate in construction of pIE1-*cspE-6H*
pIE1-*cspE-6H*	This study	CspE-6H expression under araP

1. Cherepanov PP, Wackernagel W. Gene disruption in Escherichia coli: TcR and KmR cassettes with the option of Flp-catalyzed excision of the antibiotic-resistance determinant. Gene. 1995;158(1):9-14. PubMed PMID: 7789817.
2. Lutz R, Bujard H. Independent and tight regulation of transcriptional units in Escherichia coli via the LacR/O, the TetR/O and AraC/I1-I2 regulatory elements. Nucleic acids research. 1997;25(6):1203-10. PubMed PMID: 9092630; PubMed Central PMCID: PMC146584.
3. Tabor S, Richardson CC. DNA sequence analysis with a modified bacteriophage T7 DNA polymerase. Effect of pyrophosphorolysis and metal ions. J Biol Chem. 1990;265(14):8322-8. PubMed PMID: 2159476.
4. Yosef I, Bochkareva ES, Bibi E. Escherichia coli SRP, Its Protein Subunit Ffh, and the Ffh M Domain Are Able To Selectively Limit Membrane Protein Expression When Overexpressed. mBio. 2010;1(2). Epub 2010/08/18. doi: e00020-10 [pii]

10.1128/mBio.00020-10. PubMed PMID: 20714446; PubMed Central PMCID: PMC2921155.
5. Yosef I, Bochkareva ES, Bibi E. E. coli SRP, its protein subunit Ffh and the Ffh M-domain are able to selectively limit membrane protein expression when over-expressed. mBio. 2010;doi:10.1128/mBio.00020-10
