# Supplementary material for: Model Uracil-Rich RNAs and Membrane Protein mRNAs Interact Specifically with Cold Shock Proteins in Escherichia coli
Source: PLoS One. 2015 Jul 30;10(7):e0134413. doi: 10.1371/journal.pone.0134413 (PMC4520561; doi:10.1371/journal.pone.0134413)
Supplement: S3 Table — (PDF) [file pone.0134413.s006.pdf]

Table S3. ***E. coli*** **strains.**

<table>
<tr><th>Strain</th><th>Source</th><th>Relevant genotype</th></tr>
<tr><td>BL21(DE3)pLysE</td><td>Invitrogen</td><td>6H-CspE over-expression</td></tr>
<tr><td>BW25113</td><td rowspan="3">NBRP-E.coli at NIG</td><td>Wt</td></tr>
<tr><td>BW25113 ΔcspE:kan</td><td>ΔcspE:kan. P1 donor</td></tr>
<tr><td>BW25113 ΔcspC:kan</td><td>ΔcspC:kan</td></tr>
<tr><td>BW25113 ΔcspC</td><td>This study</td><td>ΔcspC. P1 acceptor</td></tr>
<tr><td>BW25113 ΔcspCΔcspE:kan</td><td>This study</td><td>ΔcspCΔcspE:kan</td></tr>
</table>
