# Supplementary material for: Model Uracil-Rich RNAs and Membrane Protein mRNAs Interact Specifically with Cold Shock Proteins in Escherichia coli
Source: PLoS One. 2015 Jul 30;10(7):e0134413. doi: 10.1371/journal.pone.0134413 (PMC4520561; doi:10.1371/journal.pone.0134413)
Supplement: S4 Table — (PDF) [file pone.0134413.s007.pdf]

Table S4. qPCR primers.

Name	Sequence (5' NNN…NNN 3')	Use
potE_fwd	TACGGCGTCTCACTGCTGAT	IMP mRNAs (MPRs)
potE_rev	CAATCTGCACTGGCGACAAA	
aroP_fwd	GCGATAGGGACCGGGTTATT	
aroP_rev	GAAAGGCGATAAAACCAGCAA	
btuC_fwd	ACTGGATGATGGGCGGTTT	
btuC_rev	GGCCTGGACTGACAACAGATC	
cybB_fwd	CAACCCCGCCGATTATACCT	
cybB_rev	ATCACTGGCAGCGCAATAAA	
brnQ_fwd	GGTAAAGTCGCTGGCGTACTG	
brnQ_rev	TCCCCACTTCAAAGGAAACG	
pgpB_fwd	TTCCGGTCACACGATGTTTG	
pgpB_rev	CCAGACCAGCAAGATAGCAATG	
cyoB_fwd	GTTACGATCGCTGGCATTATTTT	
cyoB_rev	CGGAGGTCAGCCACTCTTTC	
dppB_fwd	CCGATGTGGCAGCAGTATCTC	
dppB_rev	GCACGAACTCTTCCCAAACC	
galP_fwd	CAGCAATTCACCGGGATGA	
galP_rev	TCACGGTCCCCCACATTT	
secG_fwd	TTCCGCTACGCTGTTTGGTT	
secG_rev	ACCCAGCACCAGACTGATGAT	
secY_fwd	AACGCCGCATTGTGGTAAAC	
secY_rev	ACCCCCGCCATATTCACTTT	
gatC_fwd	ATCGCGACGCAAACTATTGG	
gatC_rev	GAGAACCGCCCTGATCCATT	
rpe_fwd	GCCAATGGTGCTGAAATCCT	Cytoplasmic mRNA (CPRs)
rpe_rev	AGCGAAATCAGGCACAATGC	
prfA_fwd	AGGAACGTTTTCGCGCATTA	
prfA_rev	GCGGTTTCGATATCTTCCTGAA	
cysK_fwd	CGCTGGCAGGTGAAGAGATT	
cysK_rev	TGACTTTATCGACCAGCTTGAGAT	
rpoD_fwd	ATGGGCACCGTTGAACTGTT	
rpoD_rev	GATATTCAGCAACGGAGCATTG	
pgi_fwd	GATCCGGCAACGCTTGACTA	
pgi_rev	CCAGGCTGAACGGAGTGATT	
pgk_fwd	CGCGTAGCAACCGAGTTCTC	
pgk_rev	CGGAAGCATCACCGATATCC	
rplK_fwd	AACATCATGGAATTCTGCAAAGC	

rplK_rev	AAAGAACGGTCAGCGTAAACG	
RnpB_fwd	CGTACCTTATGAACCCCTATTTGG	Endogenous control
RnpB_rev	GGTGAAAGGGTGCGGTAAGA	
SsrA_fwd	TCTGGATTCGACGGGATTTG	
SsrA_rev	AGCGTAGTTTTCGTCGTTTGC	
16SrRNA_fwd	CCTGGTAGTCCACGCCGTAA	
16SrRNA_rev	CTCAAGGGCACAACCTCCAA	
23SrRNA_fwd	ATAGGTGGGAGGCTTTGAAGTG	
23SrRNA_rev	CGGATTACGGGTCAACGTTAG	
Ra_fwd	TGAGGAGCCTACTTCCCGTTT	R transcript
Ra_rev	AACAGAGAAATAGCGGCAAAAATAA	
Rb_fwd	ACCGTCACGAGCATCATCCT	
Rb_rev	CGGACGGCGTTAAAGTTGTT	
Rc_fwd	CGGTACGTTGGCAAACTCTGT	
Rc_rev	GAGGATGTAGGTGACGCAAACA	
Rd_fwd	GAAGTCTGCCCCGGTACCA	
Rd_rev	GCCCGACCTGTTTGATATGC	
